# Cocreative Development of Robotic Interaction Systems for Health Care: Scoping Review

**DOI:** 10.2196/58046

**Published:** 2024-09-12

**Authors:** Pascal Müller, Patrick Jahn

**Affiliations:** 1 Health Service Research Working Group | Acute Care, Department of Internal Medicine, Faculty of Medicine, University Medicine Halle (Saale), Martin-Luther-University Halle-Wittenberg, Magdeburger Straße 12, Halle (Saale), 06112, Germany, 49 3455574001

**Keywords:** human-robot interaction, cocreation, robotics, user-centered design, health care

## Abstract

**Background:**

Robotic technologies present challenges to health care professionals and are therefore rarely used. Barriers such as lack of controllability and adaptability and complex control functions affect the human-robot relationship. In addition to educational opportunities, the possibility of individual adaptation can improve the usability and practical implementation of robotics. Previous work has focused on developments from a technology-centered perspective and has included user interests too late in the process.

**Objective:**

This study addresses the following research question: What cocreative research approaches are used in the field of nursing robotics to improve the usability, intended use, and goal-directed application of robotic developments for nurses and to support the nursing process?

**Methods:**

This scoping review provides an overview of the topic and the research activities taking place within it. Five databases and the reference lists of the identified publications were searched for studies without further restrictions. Studies were included if they developed and evaluated interaction and control platforms for robotic systems in health care in a cocreative way with end users.

**Results:**

The search resulted in 419 hits, of which 3 publications were included. All publications were feasibility or user studies that were mainly carried out in the European Union. The 3 interaction and control platforms presented were all prototypes and not commercially available. In addition to those in need of care, all studies also included family carers and health care professionals.

**Conclusions:**

Robotic interaction and control platforms in health care are rarely, if ever, developed and evaluated with feasibility or user studies that include prototypes and end users. While the involvement of end users is crucial, this review emphasizes that all stakeholders, including health care professionals, should participate in the development process to ensure a holistic understanding of application needs and a focus on user experiences and practical health care needs. It is emphasized that the active involvement of end users in the development process is critical to effectively meeting the needs of the target group.

## Introduction

The narrative of the digital transformation of health care confronts health care professionals with the challenge of using robotic systems. The challenge is to adapt the technologies to the different and individual needs of patients. Standardized robotic functions, such as those designed for industrial robots, regularly reach the limits of their usability in care situations [1].

For a human-robot relationship to be highly usable, it must be meaningful and effective to the user. The medical journalist Nicole Janke [2] suggests that the main barriers to the use of robotics in health care are the lack of controllability, the lack of adaptability, and the complexity of control functions for changing users, contexts of use, and suitability for the user. The current inflexibility is one of the reasons for the rather low penetration of already available robotic systems in everyday life and, especially, in care. On the one hand, their use will be improved if health care professionals are introduced to the applications in a structured way through tailor-made teaching programs [3]. On the other hand, functionality will be increased if members of these nontechnical professions are given the opportunity to make certain adjustments to individual care situations themselves, thus improving situation-specific usability.

Implementation science is currently shifting from linear and safe development in controlled laboratory environments to more iterative, participatory, and complex models where interventions are developed and evaluated directly in the later field of application [4,5]. Participatory design approaches such as cocreation or design-based research [6] can be a solution to achieve usability and user acceptance. Cocreation is a collaborative approach that involves end users and relevant stakeholders in all phases of a project, from needs analysis and problem definition to the evaluation of prototypes and the final phase of a project [7].

Previous work has addressed cocreative research in technological development from a more general, theoretical perspective, in the context of raising awareness among target groups and identifying needs from a technology-centered perspective. Reference to user interests often occurs only in the testing phase of a finished technology [8-10]. Involving end users as early as possible in the development process can be seen as a way to increase acceptance and have a positive impact not only on patient satisfaction but also on the quality of care [11,12]. In addition, cocreation can increase the success of implementations of evidence-based interventions and policies through equal and deep involvement of end users [13,14]. As a result, social determinants and contextual factors responsible for the feasibility and acceptability of interventions are influenced at the earliest stage of the development cycle [15].

Currently, there are no established methods that address the adaptation of the cocreative development process to the health care or nursing context to address challenges such as collaboration or power structures [8]. To our knowledge, there also have been no systematic reviews focusing on robotic interaction and control platforms in health care. Therefore, this study addresses the following research question: What cocreative research approaches are used in the field of nursing robotics to improve the usability, intended use, and goal-directed application of robotic developments for nurses and to support the nursing process? The following subquestions can be formulated: (1) How is the cocreative process of robotic technology development designed in the context of nursing? (2) How can changes in usability, intended use, and goal-directed application be measured over the course of the development process?

## Methods

A scoping review was carried out to provide an overview of the research field, outlining the extent and nature of research activity, mapping approaches and key concepts, and identifying research gaps [16]. The content and structure of the report are based on the PRISMA (Preferred Reporting Items for Systematic Reviews and Meta-Analyses) statement [17].

### Search Strategy

**Figure 1.**
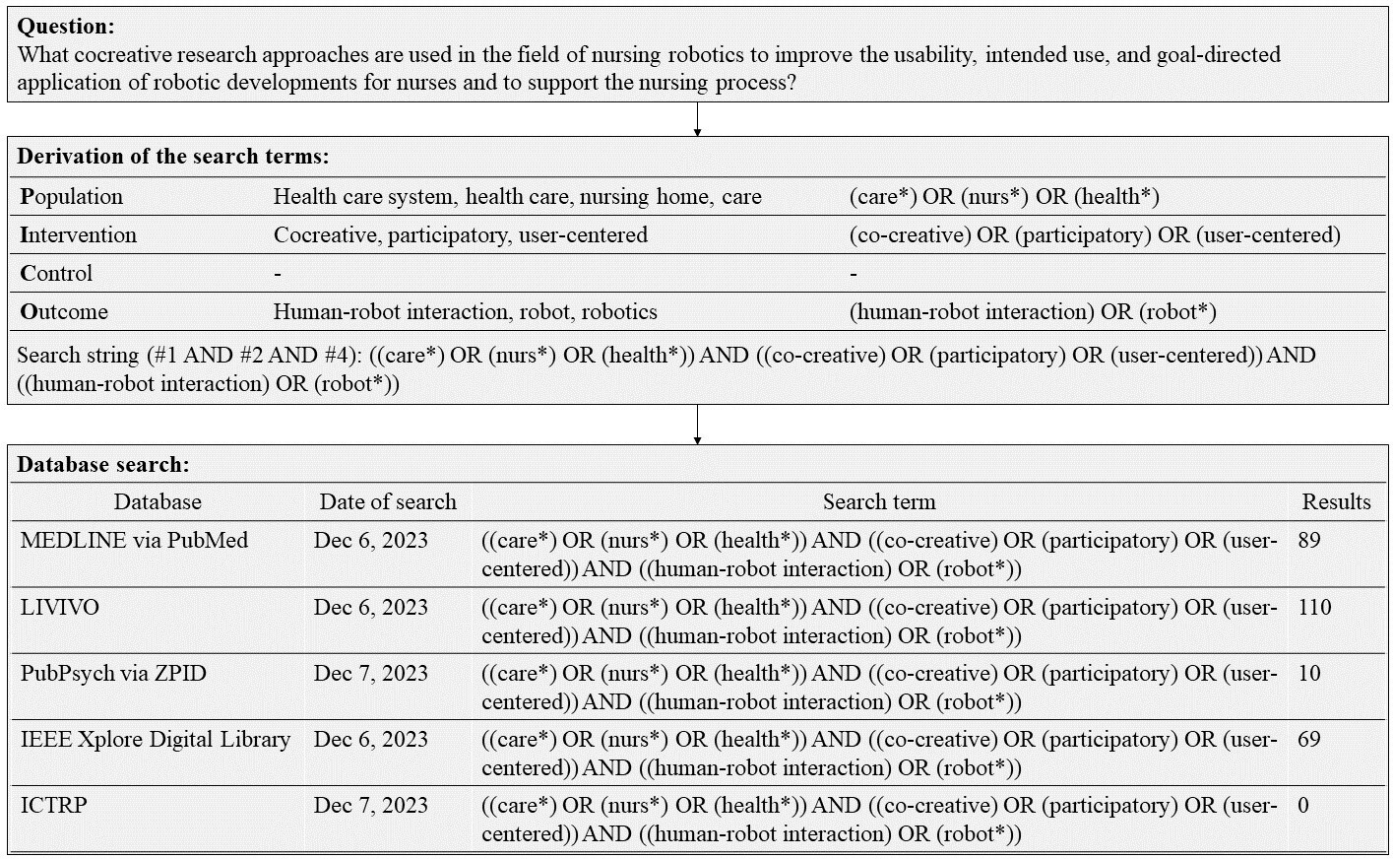
Derivation of the search terms and the database search. ICTRP: International Clinical Trials Registry Platform.

A systematic search was conducted of MEDLINE (via PubMed), LIVIVO, PubPsych (via ZPID), and IEEE Xplore Digital Library (via the IEEE website). Trial registrations were searched via the International Clinical Trials Registry Platform (ICTRP). The search terms were derived using the population, intervention, control, outcome (PICO) scheme. Figure 1 illustrates the derivation of the search terms and the database search.

The reference lists of the publications identified by the search were scanned for additional relevant publications. Only publications that reported on robotic interaction and control platforms and described a cocreative development process were included.

### Study Selection

The criteria for the selection of publications are described in Textbox 1. According to the question of this scoping review, robotic interaction and control systems that were developed in a nursing context with end users (nurses) and evaluated in practice were included.

The term *robotic system* used in this paper is based on the ISO 8373:2012 definition. According to this, a robot performs useful tasks autonomously, in the sense that it is able to perform these tasks on the basis of its sensor data without human intervention.

Despite the great progress in robotics, it remains a challenge to synthesize a variety of interaction scenarios (eg, speech, image, text, or movement) in a natural way. Research in human-robot interaction includes both multimodal input signals from humans to robots and multimodal output signals from robots to humans [18]. The aim is to improve the user experience, reduce annoying processes, and promote adoption. The latest research approaches in the field of care also need to be considered in order to translate advances in robotics into practice by developing a natural and adaptive style of interaction [19].

The relevance of a robotic intervention to health care is determined by its structure and services. For example, health services can be provided in the outpatient setting by general practitioners or specialists in nonmedical professions, in the inpatient setting, and as rehabilitation services. In addition to the treatment of diseases, prevention and health promotion are also a focus of the health care system [20,21].

As the end users are explicitly defined as nurses, robotic interventions that are more established in the medical context (eg, surgery) and are subject to different frameworks were excluded. The preliminary research identified a great deal of development of robotic systems and application scenarios for nursing care. However, as the purely technical consideration of application has not yet led to comprehensive implementation, new research approaches such as participation should be brought into focus. Even pure evaluations in cocreative design cannot do justice to the problem and were therefore excluded from this scoping review. The language of publication was not restricted to avoid further reducing the search results of relevant publications.

Textbox 1. **Inclusion and exclusion criteria of the search.**

**Inclusion criteria**

- Robotic systems, defined according to ISO 8373:2012
- Health care
- Intervention: interaction and control platform for robots
- Outcome: cocreative development and evaluation

**Exclusion criteria**

- Cocreative assessment only
- Development of robotic systems
- Intended use: medical procedures

### Collection and Analysis of Data

The characteristics of the included studies are summarized and assessed in Multimedia Appendix 1 [22-24]. The summary and preparation of the data can be described as “data-driven thematic analysis,” in which prominent and recurring themes in the literature are identified, summarized under thematic headings, and subsumed into a higher-order theoretical structure through induction and interpretation [25,26].

## Results

### Research and Study Selection

**Figure 2.**
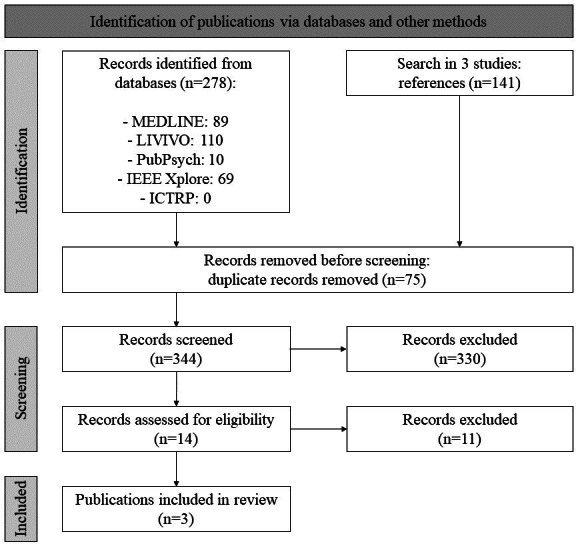
Flowchart of the study selection. ICTRP: International Clinical Trials Registry Platform.

The databases were searched manually by PM in December 2023. There were a total of 419 hits, from which 75 duplicates were removed. The remaining 344 hits were reduced to 14 full texts after title and abstract screening. After checking the full texts for eligibility, 3 studies were included. The screening process was repeated with the references of the 3 included studies. However, no further relevant sources could be identified, so 3 studies were finally included (Figure 2). Uncertainties regarding inclusion were discussed with PJ. The bibliographic data of the included publications and the full table of extracted data can be found in Multimedia Appendix 1 [22-24].

### Characteristics of the Included Studies

All publications included were in English. The studies were conducted in 6 countries; 4 of these countries were in the European Union (EU-28), which was represented by 2 publications [22,23]. Both studies were multinational and were partly conducted in Switzerland and Germany. The third included study was conducted in New Zealand [24].

All included publications were feasibility or user studies [22-24]. All had a cocreative study design. This involves users and stakeholders in the development of the robotic interaction and control platform.

One publication did not provide information about ethics committee approval and did not mention that informed consent was obtained from participants or their carers [23].

### Characteristics of the Study Participants

In addition to involving end users, cocreative development approaches also require consideration of relevant stakeholders who may interact with the end product and have a legitimate interest in its design. Health care is not an individual service provided by a single professional group, but requires interdisciplinary cooperation between different professions. In the spirit of a stakeholder analysis, the participants in the included publications are identified below.

The 3 included studies reported a total of 299 participants (Table 1), with additional carers including informal carers in one study and both formal and informal carers in another study. In one publication, the term *carer* could not be assigned to formal or informal care. In addition, one study included family members, facility managers, general practitioners, pharmacists, sociologists, geriatricians, psychologists, and computer specialists. One study involved professional teleassistants in a service center. The studies were designed in such a way that the researchers involved the carers in a cocreative way in the development of application scenarios and interaction concepts. People older than 65 years were included in 2 studies, while in 1 study the average age of the participants was only reported as 80.5 years. Children and adolescents were not reported. One study did not report the health status of the participants. The other 2 reported on people with mild to moderate cognitive impairment. One study defined intact hearing and vision as inclusion criteria (Table 1).

**Table 1.** Characteristics of the included participants (n=299). The studies included a mean of 99.67 (SD 100.5; range 16-241) participants.

Characteristics	Studies, n	References
**Age group (years)**		
≥65	2	Margaritini et al [22], Mast et al [23]
18 to 64	0	None
<18	0	None
N/A^a^	1	Tiwari et al [24]
**Health status**		
Without cognitive impairments	0	None
Cognitive impairments	2	Margaritini et al [22], Tiwari et al [24]
Without hearing or visual impairments	1	Margaritini et al [22]
N/A^a^	1	Mast et al [23]
**Other persons involved in care**		
None	0	None
Formal caregivers	0	None
Informal caregivers	1	Mast et al [23]
Formal and informal caregivers	1	Margaritini et al [22]
“Caregiver” (not further specified)	1	Tiwari et al [24]
Health care specialists	1	Tiwari et al [24]
Subject-matter experts	2	Mast et al [23], Tiwari et al [24]

a N/A: not available.

### Characteristics of the Robotic Interaction and Control Platforms

The 3 studies reported on different robotic interaction and control platforms (Textbox 2). All were developed as prototypes and were not commercially available. One study was particularly noteworthy for claiming that the platform that was developed worked for all available service robots. It took into account the dynamic autonomy of the robot and the different digital skills of the target groups. Machine learning could be used to individualize the robot and extend its range of functions [23].

The capabilities and functions of the robotic systems are retrospectively summarized in 4 themes derived from the studies after review (Table 2). Carrying or manipulating objects was described in 1 case. For example, a water bottle was picked up and brought to the user [23]. Reminder functions were described in 2 studies, including for taking medication or for upcoming appointments [22,24]. Monitoring of people was described in 2 studies, including recording of well-being and sleep quality or emergency detection and assessment [22,24]. Communication with third parties via text, audio, or video was described in all scenarios.

Two studies developed an interaction concept consisting of differentiated user interfaces with an adapted range of functions for different end users (people in need of care, carers, and health care professionals) [22,23]. The user interfaces for health care professionals were described as web-based applications in all publications.

Interaction via screens of different sizes using touch gestures was described in all 3 studies. A combination of audio output and written visualization was also chosen as output in all 3 studies. In 2 studies, facial or audio recognition of the target person was used to start the interaction [22,24]. The following requirements were defined for the user interface: a flat menu structures with step-by-step interactions [23]; no foreign or technical language [23]; large and high-contrast colors, buttons, and fonts [22-24]; and the possibility to adjust the volume [22,24].

Textbox 2. **Brief description of the robotic interaction and control platforms.**

**GUARDIAN platform [22]**

- Senior App: enables direct feedback to caregivers from the interaction
- Caregiver App: provides the ability to remotely monitor the older person’s well-being and activities
- Misty II: serves as a stress sensor for older people

**Interaction and control platform for the Care-O-bot 3 [23]**

- Care-O-bot 3: development platform
- User Interface for Local Elderly User (UI-LOC): enables autonomous activities
- User Interface for Remote Caregivers (UI-CG): enables semiautonomous navigation, scene-based autonomous manipulation without predefined action sequences, and training of objects and action sequences
- User Interface for Professional Teleassistants (UI-PRO): enables semiautonomous telemanipulation, object training, error handling, and emergency management

**Robogen [24]**

- Web-based application to support the medication process in home care

**Table 2.** Functions of the robotic systems.

Functions	Margaritini et al [22]	Mast et al [23]	Tiwari et al [24]	Studies, n
Carry or manipulate objects		✓		1
Reminders (eg, medications, appointments)	✓		✓	2
Monitoring (eg, well-being, emergencies)	✓		✓	2
Communication (eg, video telephony)	✓	✓	✓	3
Functions, n	3	2	3	

### Cocreative Study Design

Margaritini and colleagues [22] divided the cocreation process into 2 phases. In the first, 3-month phase, people with care needs and their carers (formal and informal) used an initial version of the platform. The aim of this phase was to evaluate the response of older people to the use of this new technology. As a result, technical change requests from older people were identified in order to improve the overall experience with the platform and, in particular, the usability of the graphical interface. At the beginning of the study, participants were introduced to the platform by the research team (consisting of psychologists, biomedical engineers, and physiotherapists) and helped to set it up. Monthly evaluation visits were then carried out to collect quantitative and qualitative data. The second phase followed the same participation pattern. The platform was also tested over a period of 3 months.

Mast and colleagues [23] refer to user-centered design in their research approach. A total of 6 user studies were conducted at different stages of the project. Again, 2 main phases can be identified. In the first phase (preparatory studies), mainly user studies and analytical studies were carried out. A needs analysis was carried out with older people and care staff using a mixed methods design. The focus was on the requirements for robotic systems and their integration into the daily life of end users, as well as on the technical requirements. Iterative design was the second phase. After the technical development, the existing prototypes were evaluated with older people, informal carers, and professional teleassistants. For this purpose, a usability study was carried out to evaluate the different user interfaces with representatives of the target group. The results were then used to revise the platform.

The third study, by Tiwari and colleagues [24], had 3 iterative cycles. The first cycle, which defined the framework concepts, involved developing an understanding of the underlying process that the platform was intended to map. This was done by observing nurses at work and then evaluating the information collected. In addition, interviews were conducted with people in need of care, relatives, facility management, nursing staff, doctors, and pharmacists. In the second cycle (the design of the application), prototypes of the application were discussed with computer scientists, sociologists, geriatricians, psychologists, nurses, and doctors. In the final phase of testing and refinement, a usability study was carried out with people in need of care.

The evaluation methods used by the studies (Table 3) collected both quantitative and qualitative data on requirements for robotic interventions, interactions and controls, usability, ease of use, and acceptance, using established methods such as focus groups, think-aloud, and questionnaires. Observational data were collected using video, photographs, and transcripts. Interviews were conducted without information about the use of guidelines. Recordings of the user interfaces of the robotic systems were also used for evaluation. Carers, health professionals, and experts were involved in the evaluation in addition to older people. All publications reported on established evaluation tools.

The interventions were reviewed for their characteristics. Three studies were conducted in the participants’ home environment [22-24]. In 1 study, the interaction between the participants and the robotic systems followed a schedule set by the researchers [24]. In the other 2 studies, participants were free to interact with the system [22,23].

The duration of the intervention, in terms of participants’ exposure to the robotic system or use per person, was reported in all publications. One study reported 2 intervention cycles of 3 months each [22]. The other 2 studies reported durations of 1 hour [23] and 2 hours [24].

**Table 3.** Evaluation methods.

Evaluation method		References
**Health questionnaires**		
	Short Form Health Survey	Margaritini et al [22]
	Mini-Mental State Examination	Margaritini et al [22]
	Generalized Anxiety Disorder Scale	Margaritini et al [22]
**Outcome-related measurements **		
	EQ-5D-5L	Margaritini et al [22]
	Social Connectedness Scale	Margaritini et al [22]
	Zarit Burden Interview	Margaritini et al [22]
**Evaluation of technology **		
	Unified Theory of Acceptance and Use of Technology	Margaritini et al [22]
	Technology Acceptance Model	Margaritini et al [22]
	AttrakDiff questionnaire	Mast et al [23]
**Data collection methods **		
	Think aloud	Tiwari et al [24]
	Self-created, modified, or unspecified questionnaires	Margaritini et al [22], Mast et al [23], Tiwari et al [24]
	Interviews (persons in need of care, caregivers, health care professionals, experts)	Margaritini et al [22], Mast et al [23], Tiwari et al [24]
	Video or observation logs	Mast et al [23], Tiwari et al [24]
	Robot data (touchscreen)	Tiwari et al [24]
	Focus group	Margaritini et al [22], Mast et al [23], Tiwari et al [24]
	Ethnographic study	Mast et al [23]
	Interaction analysis	Mast et al [23]
	Cognitive status (unspecified)	Tiwari et al [24]

### Outcomes

The results of the tests were not described in detail in the trials. One publication merely presented the study protocol for its development [22]. The other 2 studies focused on the presentation of the platforms and the incorporation of the results into development; as subprojects of larger studies, only selected results were presented [23,24]. Technical aspects such as feasibility, usability, suitability, functionality, and specific requests for future features were recorded as outcomes. The user perspective was considered in all 3 studies, with usefulness and interaction with the robot being of interest. Two studies described the preceding requirements analysis, which specified functions and requirements that were crucial for acceptance by the target group [23,24]. An evaluation of the cocreative collaboration between the participants was not mentioned in any of the publications.

## Discussion

This scoping review on the state of the art in cocreative development of robotic interaction and control platforms in health care shows that only a few publications have dealt with the interaction and control of robots by end users in a cocreative way. The studies have all been feasibility or user studies of prototypes with target groups including people in need of care, carers, or health care professionals. Only technical aspects such as usability or functionality were described. None of them evaluated the cocreative study design.

The identified studies all focused on home care. This takes into account the expected increasing shortage of skilled care workers. In order to reduce the workload of carers and at the same time enable people to live self-determined and independent lives for as long as possible, their own home is the ideal place [27-29]. There are no research reports on residential care.

The applicability of robotic systems in health care requires interaction and control mechanisms that can be easily adapted to the individual preferences of health care professionals without the need for engineers or programmers. In addition, this interaction and control should be based on familiar concepts such as touch-based screen inputs or audio commands. The aim should be to use robotic interventions to create ethically justifiable and socially acceptable added value that primarily supports and relieves health care professionals in their activities and accompanies those in need of care in their daily lives with dignity. The aim should not be simply to compensate for system deficits and staff shortages [30].

In the development process of such interaction and control platforms, it is important to distance oneself from purely technical solutions and instead rely on the active participation of the end users in order to reduce ethical concerns and avoid developments that do not meet the needs of the target group. All 3 studies included in this review considered the application needs and requirements of the end users in terms of “user-centered design.” To this end, a needs analysis was first carried out to understand the underlying processes and requirements of the platform. The focus was on the user interface, that is, the controls and existing technical functions. After technical implementation in the form of prototyping, these were discussed with the end users. Improvements were then made to the platform based on the data collected, and the platform was evaluated again with the target group. In this way, it was possible to design a user-oriented end application that, although not commercially available, at least addressed the acceptance problem as a possible cause of the low penetration of robotics in health care [31].

The available studies have also shown that the involvement of potential end users alone is not sufficient. All stakeholders need to be involved in order to obtain a holistic picture of the intended application. In addition to care recipients and their carers (formal and informal), implementation often has an impact on other professions, such as doctors or therapists. All legitimate stakeholders should therefore be considered and involved in the technical development.

The approach of the included studies represents a change of perspective from a purely technical consideration of functions and control elements to an orientation toward the experiences and needs of end users and practical care. The underlying development process is oriented toward users and their everyday lives as well as the care process, as required by Roland Berger GmbH [32]. The generalization of the care process into standardized procedures is a challenging and complex scenario for robotic interventions due to the human component, that is, the interests of the end user. When aspects such as individualized interactions are added, it becomes impossible for technical developers to meet the requirements and needs of the target group without the involvement of health care professionals in the development process [33,34]. Health care professionals are an important part of the research. With their understanding of diseases and their impact on the lives of those in need of care, as well as their own research expertise, they are asked as potential end users to influence technical developments toward their needs and requirements for interaction and control [35]. The generally skeptical attitude of health care professionals toward technical applications [33,34] explains why the results of this review identified only a few publications on this question, which also referred exclusively to prototypes.

The literature suggests that technical feasibility studies in cocreative design have used both quantitative and qualitative research methods. Quantitative analyses were mainly used to objectively measure the success of the study, while qualitative methods were used selectively and for a specific topic (eg, needs analysis or evaluation of prototypes). The studies did not discuss evaluation of the methodology or presentation of results and processes. Therefore, no comparison with other development concepts is possible. It remains questionable how the stakeholders involved perceived the collaboration and whether the eventual acceptance of the platforms was higher than with previously developed platforms. An evaluation of the attitudes of the health care professionals involved in the studies toward technological interventions could provide additional valuable insights for the implementation of technologies in health care.

The scoping review method used here is only intended to provide an overview of the research field. It does not consider the effectiveness of the studies or derive recommendations for clinical practice [36]. So far, only feasibility studies of robotic interaction and control platforms are available. A systematic assessment of the included studies using a critical appraisal tool was not carried out. This was not part of the research interest.

The operationalization of the research question and the development of the search terms are justified and can be used as a starting point for further literature searches. The process is transparent and fully documented (Figure 1). Due to the resources available in the project, only one reviewer was involved in the selection and assessment of the studies. Despite the care taken, bias cannot be excluded.

## Supplementary material

10.2196/58046 Multimedia Appendix 1 Data on included publications.

10.2196/58046 Checklist 1 Preferred Reporting Items for Systematic reviews and Meta-Analyses extension for Scoping Reviews (PRISMA-ScR) checklist.

## Abbreviations

EU: European Union
ICTRP: International Clinical Trials Registry Platform
PICO: population, intervention, control, outcome
PRISMA: Preferred Reporting Items for Systematic Reviews and Meta-Analyses

## References

[1] Janda V, Schubert C, Schulz-Schaeffer I (2019). Berliner Schlüssel Zur Techniksoziologie.

[2] Janke N (2018). Robotik in der Pflege. Verband der Ersatzkassen.

[3] Geist L, Immenschuh U, Jahn P (2022). Identifikation von lernfördernden maßnahmen zur einführung von digitalen und assistiven technologien (DAT) in prozesse der pflegerischen versorgung: eine qualitative studie. HB Science.

[4] Verloigne M, Altenburg T, Cardon G (2023). Making co-creation a trustworthy methodology for closing the implementation gap between knowledge and action in health promotion: the Health CASCADE project. Perspect Public Health.

[5] Gibbons M (2000). Mode 2 society and the emergence of context-sensitive science. Sci Pub Pol.

[6] Armstrong M, Dopp C, Welsh J, Kimmons R, Caskurlu S (2020). The Student’s Guide to Learning Design and Research.

[7] Vargas C, Whelan J, Brimblecombe J, Allender S (2022). Co-creation, co-design, co-production for public health - a perspective on definition and distinctions. Pub Health Res Pract.

[8] Dalko K, Kraft B, Jahn P, Schildmann J, Hofstetter S (2023). Cocreation of assistive technologies for patients with long COVID: qualitative analysis of a literature review on the challenges of patient involvement in health and nursing sciences. J Med Internet Res.

[9] Kehl C, Bendel O (2018). Pflegeroboter.

[10] Maibaum A, Bischof A, Hergesell J, Lipp B (2022). A critique of robotics in health care. AI Soc.

[11] Lyon AR, Munson SA, Renn BN (2019). Use of human-centered design to improve implementation of evidence-based psychotherapies in low-resource communities: protocol for studies applying a framework to assess usability. JMIR Res Protoc.

[12] Dopp AR, Parisi KE, Munson SA, Lyon AR (2020). Aligning implementation and user-centered design strategies to enhance the impact of health services: results from a concept mapping study. Impl Sci Commun.

[13] Flaspohler PD, Meehan C, Maras MA, Keller KE (2012). Ready, willing, and able: developing a support system to promote implementation of school-based prevention programs. Am J Community Psychol.

[14] Greenhalgh T, Jackson C, Shaw S, Janamian T (2016). Achieving research impact through co-creation in community-based health services: literature review and case study. Milbank Q.

[15] Longworth GR, Goh K, Agnello DM (2024). A review of implementation and evaluation frameworks for public health interventions to inform co-creation: a Health CASCADE study. Health Res Policy Syst.

[16] Arksey H, O’Malley L (2005). Scoping studies: towards a methodological framework. Int J Soc Res Methodol.

[17] Moher D, Liberati A, Tetzlaff J, Altman DG, PRISMA Group (2009). Preferred Reporting Items for Systematic reviews and Meta-Analyses: the PRISMA statement. PLoS Med.

[18] Su H, Qi W, Chen J, Yang C, Sandoval J, Laribi MA (2023). Recent advancements in multimodal human-robot interaction. Front Neurorobot.

[19] Fang B, Wei X, Sun F, Huang H, Yu Y, Liu H (2019). Skill learning for human-robot interaction using wearable device. Tinshhua Sci Technol.

[20] (2020). Das Deutsche gesundheitssystem. Bundesministerium für Gesundheit.

[21] (2018). Gesundheitsversorgung in Deutschland. Institut für Qualität und Wirtschaftlichkeit im Gesundheitswesen (IQWiG).

[22] Margaritini A, Benadduci M, Amabili G (2022). The social robot companion to support homecare nurses: the guardian study protocol. Contemp Clin Trials Commun.

[23] Mast M, Burmester M, Kruger K (2012). User-centered design of a dynamic-autonomy remote interaction concept for manipulation-capable robots to assist elderly people in the home. J Hum Robot Interact.

[24] Tiwari P, Warren J, Day K (2011). Empowering older patients to engage in self care: designing an interactive robotic device. AMIA Annu Symp Proc.

[25] Dixon-Woods M, Agarwal S, Jones D, Young B, Sutton A (2005). Synthesising qualitative and quantitative evidence: a review of possible methods. J Health Serv Res Policy.

[26] Noblit GW, Hare RD (1988). Meta-Ethnography: Synthesizing Qualitative Studies.

[27] Klie T, Storm A (2017). Pflegereport 2017 Gutes Leben Mit Demenz: Daten, Erfahrungen Und Praxis.

[28] Afentakis A, Maier T (2010). Wirtschaft Und Statistik.

[29] Mestheneos E (2011). Ageing in place in the European Union. Glob Age Iss Act.

[30] Zegelin A, Meyer G (2018). Roboter gegen personalengpässe in der pflege?. Pflege.

[31] Hülsken-Giesler M, Brandenburg H, Dorschner S, Bekel G, Fenchel V, Georg J (2015). Pflegewissenschaft 1 Lehr- Und Arbeitsbuch Zur Einführung in Das Wissenschaftliche Denken in Der Pflege.

[32] (2017). ePflege: informations- und kommunikationstechnologie für die pflege. Roland Berger.

[33] Papadopoulos I, Koulouglioti C, Lazzarino R, Ali S (2020). Enablers and barriers to the implementation of socially assistive humanoid robots in health and social care: a systematic review. BMJ Open.

[34] Huang R, Li H, Suomi R, Li C, Peltoniemi T (2023). Intelligent physical robots in health care: systematic literature review. J Med Internet Res.

[35] Paulicke D, Helbig K, Voigt J, Stoevesandt D, Jahn P Multimodale und interprofessionelle weiterbildungsangebote für ärzte und pflegefachkräfte zur integration technologischer rund robotischer assistenz zur versorgungssicherung für ältere menschen mit demenz in Sachsen-Anhalt.

[36] Grant MJ, Booth A (2009). A typology of reviews: an analysis of 14 review types and associated methodologies. Health Info Libr J.

